# Supplementary material for: Conditional chemoconnectomics (cCCTomics) as a strategy for efficient and conditional targeting of chemical transmission
Source: eLife. 2024 Apr 30;12:RP91927. doi: 10.7554/eLife.91927 (PMC11060718; doi:10.7554/eLife.91927)
Supplement: Figure 3—source data 3. [file elife-91927-fig3-data3.zip › Figure 3 source data3 part 1.zip!/Figure 3-Source data 3-part1/1067-9-CRISPResso_Report_c33kf3u3.zip!/CRISPResso_on_1067_9/9.Alleles_frequency_table_around_sgRNA_agccaggtgtcgcagacgat.pdf]

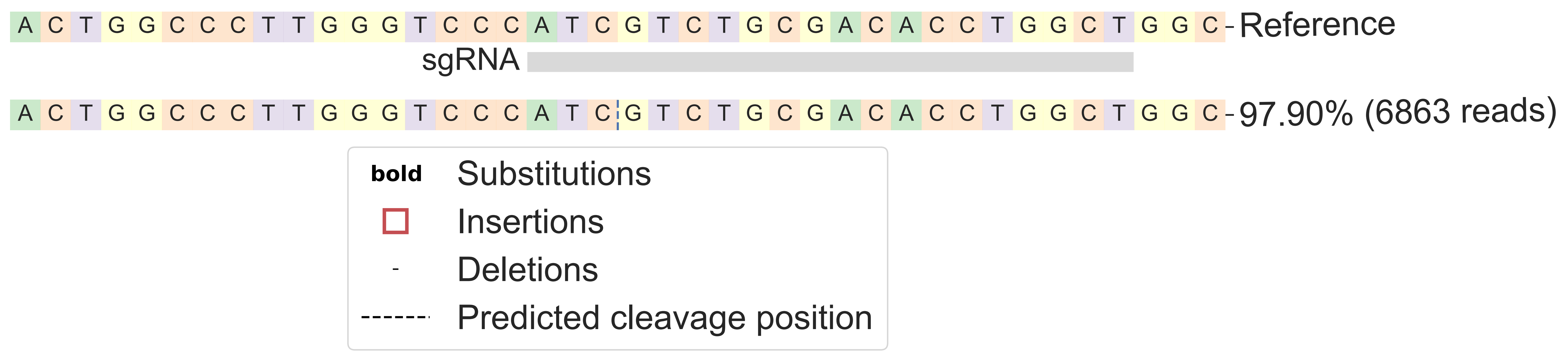
ACTGGCCCTTGGGTCCCATCGTCTGCGACACCTGGCTGGC - Reference
sgRNA
ACTGGCCCTTGGGTCCCATCGTCTGCGACACCTGGCTGGC - 97.90% (6863 reads)
bold Substitutions
Insertions
- Deletions
Predicted cleavage position
